# Larval Exposure to Chlorpyrifos Affects Nutritional Physiology and Induces Genotoxicity in Silkworm *Philosamia ricini* (Lepidoptera: Saturniidae)

**DOI:** 10.3389/fphys.2016.00535

**Published:** 2016-11-15

**Authors:** Moni K. Kalita, Kishor Haloi, Dipali Devi

**Affiliations:** Seri-Biotech Unit, Life Science Division, Institute of Advanced Study in Science and TechnologyGuwahati, India

**Keywords:** chlorpyrifos, eri silkworm, digestive enzymes, histology, genotoxicity, apoptosis

## Abstract

Chlorpyrifos is a most widely used organophosphate insecticide because of its cost effectiveness and degradable nature. However, this pesticide enters and contaminates the environment either by direct application, spray drifts or crop run off and shows adverse effect on the non-targeted organisms. *Philosamia ricini* (eri silkworm), one of the most exploited, domesticated and commercialized non mulberry silkworm is known for mass production of eri silk. The silkworm larvae get exposed to pesticide residues on the leaves of food plants. The present study investigates the effect of commercial formulation of chlorpyrifos (Pyrifos-20 EC) on eri silkworm. Initially the LC_50_ value of chlorpyrifos was determined at 24–96 h and further experiments were carried out with sub lethal concentrations of the chlorpyrifos after 24 h of exposure period. The potential toxicity of chlorpyrifos was evaluated as a fuction of metabolism and nutritional physiology in 3rd, 4th, and 5th instar larvae. Alteration in histoarchitecture of 5th instar eri silkworm gut exposed to sub lethal concentration of chlorpyrifos formulation was also studied. Chlorpyrifos induced genotoxicity in silkworm hemocytes was also investigated by single cell gel electrophoresis, micronuclei assay, and apoptosis assay. Herein, LC_50_ values of chlorpyrifos were calculated as 3.83, 3.35, 2.68, and 2.35 mg/L at 24, 48, 72, and 96h respectively. A significant decrease in trehalose activity along with digestive enzyme activity was observed in chlorpyrifos affected groups (*P* < 0.05). Further, genotoxicity study revealed higher tail percentage, tail length and tail moment of the damage DNA in chlorpyrifos exposed groups (*P* < 0.001). Moreover, at 2.0 mg/L concentration, ~10 fold increases in tail length was observed as compared to the control. Results showed activation of caspase activity following 24 h chlorpyrifos exposure (1.5 and 2.0 mg/L) in a dose-dependent manner. Moreover, in control group less number of apoptotic cells was detected, however in both chlorpyrifos exposed groups' numbers of apoptotic cells were statistically higher (*P* < 0.001). Taken together, this study provides evidence that chlorpyrifos pollution might have adverse effect on overall nutritional physiology and genotoxicity of eri silkworm that could lead to reduced survivability of this economically beneficial insect.

## Introduction

Pesticides are extensively used as pest control agents and considered as the potential mutagens in insects (Bolognesi, 2003). The use of pesticides sounds beneficial to mitigate pest problem in agricultural practices. However, these are the most common contaminants, which affect every ecosystem starting from the soil, air to water and also non-target organisms (Altinok et al., 2012; Gill et al., 2012; Wagner-Schuman et al., 2015). It is also reported that over 98% of sprayed pesticides and 95% of herbicides affect the non-targeted species (Miller, 2004). Chlorpyrifos (chlorpyrifos) [O,O-diethyl-O-(3,5,6-trichloro-2-pyridyl)-phosphorothioate], one of the broad spectrum organophosphate pesticides used both in agriculture and household pest control agents due to its non-carcinogenic and non-teratogenic nature (Charpentier et al., 2014). It acts as acetylcholine esterase inhibitor and targets the nervous system of the insects (Yin et al., 2009; Nguyen et al., 2015). Moreover, chronic toxicity of chlorpyrifos lead to reduced survival rate, adult body weight and reproductive failure of non-target fish species (Corbin et al., 2009; Eddins et al., 2009; Richendrfer et al., 2012). Additionally, alteration of biochemical contents including protein content, enzyme activity and induced genotoxicity was also observed on chlorpyrifos exposure in different non-target organisms (Kavitha and Rao, 2008; Ali et al., 2009). Chlorpyrifos is a cost efficient, degradable pesticide, hence despite of its toxicity it is extensively used since its introduction in 1965. Therefore, Chlorpyrifos has been reported as one of the commonly used organophosphate pesticide (Kiely et al., 2004; John and Shaike, 2015). However, this pesticide enters and contaminates the environment either by direct application, spray drifts or crop run off and shows adverse effect on the non-targeted wildlife populations starting from microorganism to higher vertebrates (Watson et al., 2014; Ahmad et al., 2015; Jin et al., 2015; John and Shaike, 2015; Pascotto et al., 2015).

In this regard, silkworms are no exception from this pesticide hazard as many problems appeared in sericulture industry is due to application of pesticides (Etebari et al., 2007). The silkworm larvae get exposed to pesticide residues on the leaves of food plants leading to adverse effects on silk production. Recently, silk industry in China was severely affected by organophosphate pesticides, resulting 30% decrease in raw silk annual production. The silkworm *Bombyx mori* (*B. mori*) is highly sensitive to most of the pesticides and affected by organophosphates and other insecticides (Peng et al., 2011). Zhang et al. (2008) studied the acute toxicity of various organophosphate and pyrethroid pesticides to *B. mori* and suggested controlled use of these pesticides in nearby agricultural area for sustainable development of the sericulture industry.

*Philosamia ricini* (eri silkworm), one of the most exploited, domesticated and commercialized non mulberry silkworm is known for mass production of eri silk. This silk has high demand due to its high thermal property, strength, durability and can be blended with many other materials for textile and non-textile applications. Eri silkworm is mainly cultivated in the North Eastern (N. E.) states of India (including Assam) contributing 65% of total silk production in India. Eri rearing is a peripheral activity of households scattered around the state for their livelihood. It mainly feeds on castor leaves (*Ricinus communis*), and the host plant quality and pesticide contamination are likely to affect the silk production. Although the average of pesticide application in N.E. India is not so intensive (6.13 g/Hectare), the eri silk rearing is exposed to heavy doses of pesticides, which are in close vicinity of tea estate. Thus, these pesticides might accumulate on non-target castor leaves due to spray drift process.

Although earlier reports demonstrated the effects of pesticides on *B. mori* health and physiology however, effect of pesticide contamination on the eri silkworm is still unexplored. Therefore, current study aimed to evaluate the acute toxicity of chlorpyrifos on eri silkworms. Herein, mortality rate was determined by calculating LC_50_ value of chlorpyrifos. The sub lethal effect of chlorpyrifos on behavioral changes, biochemical, and physiological alteration were investigated. Additionally, the induced genotoxicity of chlorpyrifos on eri silkworm was also studied and particularly focused on single-cell gel electrophoresis (comet assay) and micronuclei assay in order to determine the extent of DNA damage in hemocytes of eri silkworm after exposure to chlorpyrifos. Further, chlorpyrifos induced apoptosis was also taken into consideration to elucidate the probable mechanism of DNA fragmentation.

## Materials and methods

### Silkworm

Disease-free laying of eri silkworms were obtained from Mangaldoi Seri culture farm of Assam. The silkworms were reared at Seri-biotech laboratory of IASST under recommended condition at 25–27°C, 75 ± 5% relative humidity and 12: 12 (L: D) h photoperiod. The larvae were fed with *Ricinus communis* (castor) leaves harvested from the garden of IASST campus.

### Chemicals

A commercial formulation of chlorpyrifos (O,O-diethyl-O-(3,5,6-trichloro-2-pyridyl)-phosphorothioate, active ingredient 20 g in 100 ml) Pyrifos-20 EC was purchased from Assam fertilizer house, Guwahati, Assam. A stock solution of 100 mg/L was prepared by dissolving 0.05 ml of Pyrifos- 20 EC in 99.95 ml of acetone. For toxicity determination, subsequent dilutions were made with distilled water. The concentrations were calculated on the basis of the active ingredients present in the pesticide. As acetone is known to be less toxic to silkworm than other solvents and commonly used in pesticide assays (Burchfield et al., 1952; Reddy et al., 1991; Nath, 2002), therefore the same was used in the present study. All other chemicals used in this study were of analytical grade.

### Acute toxicity bioassay

Acute toxicity in eri silkworm was determined by a semi-static method in laboratory condition as per standard protocol (Zhang et al., 2008). The experiment was performed with 0.5–5.0 mg/Lchlorpyrifos (0.5, 0.8, 1.0, 1.2, 1.4, 1.6, 1.8, 2.0, 2.2, 2.4, 2.6, 2.8, 3.0, 3.2, 3.4, 3.6, 3.8, 4.0, 4.2, 4.4, 4.6, 4.8, 5.0 mg/L) concentration and repeated thrice with three replication (9 replication) for each concentration. Fifty gram of fresh castor leaves were sprayed with each freshly prepared working concentration of chlorpyrifosand air dried leaves (10 min) were fed to the pre-starved (8 h) 5th instar healthy larvae. A control set was also maintained from the same stock under the same environment and fed with distilled water sprayed castor leaves (Nwani et al., 2010). The mortality was recorded for 24–96 h exposure period and the dead larvae were removed. Behavioral changes like feeding, movement and any kind of abnormal behavior was observed and recorded in regular intervals after pesticide exposure. The LC_50_ value that would kill 50% of silkworm at different time interval i.e., 24–96 h was determined from obtained data by the method of probit analysis (Finney, 1971), and followed by selection of two sub lethal doses of chlorpyrifos for further study. All the experiments were performed three times in triplicates (50 larvae per replication).

### Metabolites

The larvae were acclimatized in laboratory condition upto 2nd instar larval stage and biochemical experiments were conducted from 3rd instar to 5th instar larval stages. All the biochemical experiments were performed three times in triplicates (*n* = 10). Total soluble protein was estimated in control and pesticide treated groups (1.5 and 2.0 mg/L) after 24 h exposure by using standard protocol of Lowry et al. (1951). Briefly, 10% tissue homogenate was prepared and protein was precipitated by adding 10% trichloroacetic acid (TCA). After precipitation, the sample was kept for 30 min at low temperature followed by centrifugation at 6000 rpm for 10 min. Further, the residue was dissolved in 0.1 N NaOH. Later1 ml of aliquots was pipette out, solution C (98 ml of 2% sodium carbonate solution mixed with 2 ml of 0.5% copper sulfate in 1% potassium sodium tartrate solution) was added and kept for 10 min. After incubation folin phenol reagent was added and kept for 30 min for color generation. A blue color was developed, which was measured at 640 nm absorbance in multimode reader (Thermo Scientific Varioskan).

Further, trehalose concentration was measured by anthrone method (Wyatt and Kalf, 1957). Herein, 10% tissue homogenate was deproteinized with 70% ethanol (1:2) and kept at 4°C for 20 min. The sample was centrifuged at 6200 rpm for 20 min and the clear supernatant was used for determination of trehalose concentration. Later, 1 ml of aliquot was added to 3 ml of anthrone reagent (2 g anthrone in 85% of ice cold H_2_SO_4_) and vortexed for 30 s. The samples were kept into boiling water bath at 100°C for 15 min and OD was measured at 620 nm.

Total soluble lipid was estimated by the method of Barnes and Blackstock (1973). Briefly, 10% tissue homogenate was prepared in chloroform: methanol mixture (2:1v/v) and centrifuged at 4000 rpm for 20 min. one milli liter of supernatant was taken, evaporated in a warm water bath and continued until drying. The tubes were left for 3 min, 2 ml of 98% H_2_SO_4_ was added and placed in warm water bath for 10 min. Further, 5 ml of 1% phospho-venilin was added, incubated for 15 min at 37°C and absorbance was taken at 540 nm.

### Enzyme assay

For enzyme assay the larvae were acclimatized in laboratory condition upto 4th instar larval stage and then exposed to sub lethal concentration of pesticide (1.5 and 2.0 mg/L). All the experiments for enzyme assay were performed three times in triplicates (*n* = 10). The whole gut of mature 5th instar larvae was isolated after 24 h chlorpyrifos exposure, rinsed three times in 1X phosphate buffer saline (pH 7.4) and cut into foregut, midgut and hindgut. For crude enzyme preparation the different gut sections were homogenized in 500 μl of sodium acetate buffer (0.1 M, pH 5.6) on ice and centrifuged at 15,000 rpm for 15 min at 4°C. In addition, control was prepared by adding the same volume of 0.1 M sodium acetate (Blakemore et al., 1995).

#### Cellulase assay

For cellulase assay, 200 μl sodium acetate buffer (0.1M, pH 5.6) was mixed with the substrate (20 μl of 0.1% sodium carboxymethyl cellulose) followed by addition of crude enzyme (50 μl) and incubation in a water bath at 37°C for 30 min. Further, 90 μl of dinitro salicylic acid solution (1.6 g sodium hydroxide, 30 g sodium potassium tartarate, 1 g 3, 5–dinitrosalicylic acid per 100 ml H_2_O) was added, and the mixture was boiled for 15 min. Glucose production was detected at 540 nm by taking the absorbance using multimode reader. The standard curve of glucose was used, and the activity was measured as μg glucose release per 30 min (Bernfeld, 1955; Weidlich et al., 2013).

#### Amylase assay

Amylase enzyme activity was measured by standard protocol (Bernfeld, 1955; Weidlich et al., 2013). Twenty micro liter of 1% glucose was mixed with 200 μl sodium acetate buffer (0.1M, pH 5.6) and followed by addition of 50 μl of crude enzyme. The mixture was incubated at 37°C for 30 min. After incubation, 90 μl of dinitro salicylic acid solution was added; the mixture was boiled for 15 min and cooled at room temperature. Maltose production was detected at 620 nm by taking the absorbance using multi-mode reader. The standard curve of maltose was prepared, and activity was measured as μg maltose per 30 min.

#### Protease assay

For protease enzyme activity, 30 μl of crude extract was mixed with 600 μl of1% casein and incubated for 30 min at 37°C. The reaction was stopped by adding 270 μl of 20% TCA (v/v). The reaction mixture was kept for 20 min in ice bath followed by centrifugation at 5000 rpm for 10 min and absorbance was measured at 280 nm. A standard curve of L-tyrosine was prepared and used for estimation (Faulk et al., 2007).

#### Lipase assay

Lipase enzyme activity was studied by adopting the method of Faulk et al. (2007). For this study, 4 nitrophenylpalmitate (0.35 mM) in Tris-HCl (0.5 mM, pH 7.4) was used as substrate. Further, 30 μl of the extract was mixed with the substrate, sodium taurocholate (6 mM), and NaCl (1 M). A standard curve of nitrophenol was prepared, and nitrophenol generation was measured at 400 nm.

#### Alanine aminotransferase (ALT) assay

ALT assay was performed following the method of Inagaki et al. (2012). Ten milli liter Tof the supernatant was mixed with 550 μl of the reaction mixture (0.5 M L-alanine, 0.2 mM NADH, 1.3 U/ml lactate dehydrogenase and 0.9 mg/ml of BSA) and vortexed. Later, 50 μl of 180 mM 2-oxoglutarate solution was added to the reaction mixture and incubated for 90 min at 30°C. Absorbance was measured at 339 nm, and the activity was expressed in unit. (1U defined as change in absorbance of 0.001 per minute per μl of enzyme sample).

### Histopathology study

The histopathological changes in the gut of 5th instar larva were studied after 24 h exposure to sub lethal concentration of chlorpyrifos (1.5 mg/L and 2.0 mg/L). Histological experiment was performed three times in triplicates (*n* = 10). The gut was removed and fixed in the Carnoy's fluid (absolute alcohol: chloroform: glacial acetic acid = 6:3:1). The histological slides were prepared by the standard protocol of dehydration and paraffin embedding (Gurr, 1959). Sections of 5 μm were cut and stained with hematoxylene and eosin. Afterwards, the sections were observed under phase contrast microscope (Zeiss Axio Cam ERC 5S).

### Genotoxicity assay

#### Measurement of hemocyte viability

The 5th instar hemolymph was collected in ice-cold tubes after 24 h of exposure to sub lethal concentrations of chlorpyrifos. Ten micro liter of hemolymph sample was mixed with 0.1% trypan blue (1:1 v/v) and observed under microscope immediately (Phillips, 1973). The numbers of trypan blue-negative and -positive cells were counted by cytometer and samples with 95% cell viability were processed for further assay. The assay was performed three times in triplicates (*n* = 10).

#### Micronuclei assay

The micronucleus test was performed by following the procedure referred by Siu et al. (2004). Hemolymph was collected from the control and pesticide exposed (after 24 h) 5th instar larvae by cutting off the prolegs. The assay was performed five times in triplicates (*n* = 5). A thin smear of hemolymph was prepared, and the slides were air-dried at room temperature in dust and moisture free environment. Further, fixation was done by dipping in absolute methanol for 15 min and air-dried for 1 h. The slides were stained in 10% Giemsa stain in 1X phosphate buffer for 30 min, washed with distilled water and dried overnight. The slides were mounted with DPX and observed under phase contrast microscope (ZeissAxio Cam ERC 5S). Two hundred cells per slide were counted with five replications. The percentage of micronuclei cells was calculated by using the following formula.

$$\begin{array}{l} \text{Micronuclei formation (\%)}\\ =\text{Number of micro nucleated cells/Total number of cells}\times 100 \end{array}$$

#### Alkaline single cell gel electrophoresis

The genotoxicity was assessed by adopting the procedure of Singh et al. (1988). At the end of the exposure period of 24 h, 10 larvae of 5th instar were selected from experimental groups (1.5 mg/L and 2.0 mg/L). A positive control was set by injecting 10 μl of 10% H_2_O_2_ to the silkworms and allowed them to feed for 24 h. The assay was performed three times in triplicates (*n* = 10). The hemolymph was collected by cutting one of the prolegs. Cell viability was tested by standard Trypan-blue exclusion method (Phillips, 1973) and samples with 95% cell viability were processed for single cell gel electrophoresis (SCGE). Fifty μl of 1% agarose in double distilled water (DDW) was smeared on one end frosted slide and air dried. The second base layer coat was given by placing 200 μl agarose (1% in PBS, pH-7.4) on the slide. About 20 μl cell suspension were mixed with 80 μl low melting point agarose (1% in PBS) and layered in the previously coated slide. Further, a third layer of 100 μl of 1% low melting point agarose was applied and covered with a cover glass. After solidification of the gel, the slide was immersed in lysing solution (2.5 M NaCl, 100 mM Na_2_ EDTA and 10% dimethyl sulphoxidein DDW and pH was adjusted to 10). one percent Triton-X-100 was freshly added and kept overnight at 4°C. The slides were placed in electrophoresis buffer (10 N NaOH, 200 mM EDTA, 10% dimethyl sulphoxide) for DNA unwinding for 20 min. Electrophoresis was carried out at 120 mV for 20 min at 4°C in dark condition. The slides were immersed in the neutralizing solution (400 mMTris, pH 7.5) for 20 min. The slides were stained with 75 μl ethidium bromide and the excess stain was removed by rinsing the slides in DDW. DNA damage was visualized by observing the slides on 40x fluorescence microscope (Leica DMI3000B). Visual scoring of the comet cells was done by ImageJ (NIH) software to analyze the DNA damage percentage. The comet cells of control hemocytes with intact round nucleus determined as head of the comet. The treated cells had a head along with a tail. Total length, tail length and head length was calculated by ImageJ (NIH) software. Further head percentage was calculated by total length minus tail length and multiplied by 100. Tail percentage was calculated by 100 minus head percentage. Tail moment was calculated by using the following formula.

$$\begin{array}{l} \text{Tail moment}=\text{Tail length}\times \text{Tail percentage}/100 \end{array}$$

#### Caspase activation assay

Caspase activation was observed by using Image-iT Live Green Caspase-3 and −7 Detection Kit (I35106, Molecular Probes, Invitrogen). Initially the 5th instar larvae were orally administered with sub lethal concentrations of chlorpyrifos and after 24 h of exposure hemolymph was collected. This assay was performed three times in triplicates (*n* = 10). The hemocytes were precipitated by centrifuging at 4000 rpm for 5 min and the precipitated cells were suspended in insect physiological saline (150 mM NaCl, 5 mM KCl, and 1 mM CaCl_2_). A uniform smear of cell suspension was prepared on a glass slide and thereafter the slide was incubated at 27°C for 15 min in an incubator (Labtech LBI-150 E, Korea). After incubation a sufficient amount of 30 fold diluted FLICA reagent was added to the cell smear and the cells were incubated for 60 min in dark condition under existing environment. Thereafter, the reagent was removed and the cells were gently rinsed with PBS. After washing sufficient amount of 1 mM Hoechst 33,342 and 5 mM propidium iodide stain was added to carry out the nuclear counter stain and incubated for 10 min under existing condition. Thereafter, the cells were washed twice with 2 mL of 1X wash buffer and analyzed immediately under a fluorescence microscope (Leica DMI3000B). The excitation/emission filter was 488/520, 350/461, and 490/635 nm for FLICA, Hoechst and propidium iodide respectively.

#### Annexin V affinity assay for detection of apoptotic hemocytes

Apoptotic hemocytes were detected by Alexa Fluor® 488 annexin V Apoptosis Kit with Alexa® Fluor 488 annexin V and PI (Molecular probes, Invitrogen, V13241). Fifth instar larval hemocytes from both control and chlorpyrifos exposed groups were collected by cutting one of the prolegs. This assay was performed three times in triplicates (*n* = 10). A smear of hymolymph (50 μl) was prepared on a glass slide and air dried for 10 min. Thereafter, 5 μL Alexa FluorR 488 annexin V (Component A) and 1 μL 100 μg/ml of propidium iodide working solution was added to the smear and incubated at room temperature for 15 min. After incubation 400 μl 1 X annexin-binding buffer was added, mixed gently and the slides were analyzed as soon as possible in fluorescence microscope (Leica DMI3000B) at 530/488 nm.

### Statistical analysis

Probit analysis was performed with Biostat (2009) software package with three times in triplicates for LC_50_ value calculation (*n* = 50). All the other experiments were performed three times in triplicates (*n* = 10) except micronuclei assay which was performed five times in triplicates (*n* = 5) and results are considered as mean ± standard deviation. One way analysis of variance (ANOVA) was carried out for statistical analysis with *P* < 0.05, *P* < 0.01, and *P* < 0.001, which was considered significant and highly significant respectively. Further, means were compared by *post hoc* Tukey's test. The graphical representations were performed using Microcal Origin version 6.1.

## Results

### Intoxication symptoms of silkworm

Intoxication symptoms body blackening, less feeding and vomiting was observed in silkworm larvae after exposure to chlorpyrifos. Oozing out of hemolymph from rectal protrusion of the larva was also recorded within 12 h of exposure to a high dose of pesticide (5 mg/L). In contrast, silkworms fed on distilled water sprayed castor leaves, showed no visible sign of toxicity as compared to pesticide affected groups.

### Determination of LC_50_

**Figure 1:**
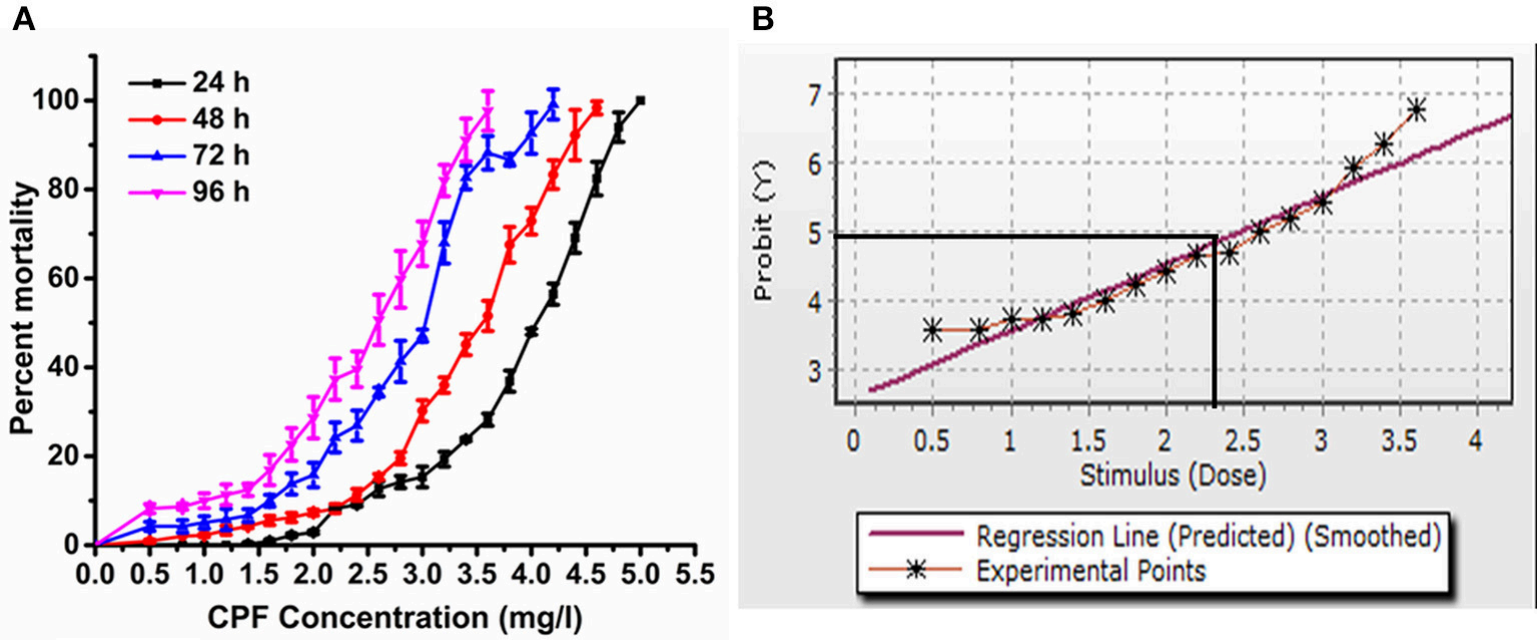
**(A)** Mortality percentage of *P. ricini* to different concentrations of chlorpyrifos at 24, 48, 72, and 96 h, and **(B)** probit analysis graph for 96 h exposure period, the experiment was performed three times in triplicates (*n* = 50).

For the experimental concentrations, results revealed that percent mortality increased sharply with increased concentration of chlorpyrifos (Figure 1A). The acute toxicity of chlorpyrifos to the silkworms was calculated by Probit analysis at different time intervals as shown in Table 1. The calculated LC_50_ values of chlorpyrifos were as 3.83, 3.35, 2.68, and 2.35 mg/L at 24, 48, 72, and 96 h respectively (Table 1, Figure 1B). Two sub lethal concentrations of chlorpyrifos (1.5 mg/L and 2.0 mg/L) were selected based on LC_50_ value of 24 h for further study.

**Table 1:** **Acute toxicity response of *P. ricini* against chlorpyrifos at different time intervals (Biostat, 2009 software package)**.

**Time (h)**	**Sample Size**	**Log 10**	**Slope ± SE**	**LC_50_value (mg/L)**	**95% CL**	**Chi square**	***P*-value**
24	50	0.58	0.8 ± 0.09	3.83	3.65 ~ 4.03	11,554	0
48	50	0.52	1.65 ± 0.61	3.35	2.51 ~ 5.42	6882	0
72	50	0.43	2.74 ± 0.51	2.68	1.79 ~ 4.02	1315	0
96	50	0.37	3.53 ± 0.22	2.35	1.95 ~ 2.95	90	0

The experiment was performed three times in triplicates (n = 50).

### Biochemical studies

The biochemical constituents are measured in control and pesticide treated groups (1.5 and 2.0 mg/L) of eri silkworm after 24 h of exposure and results are presented in Table 2. In control batch, the trehalose, protein and lipid concentrations were increased from 3rd instar to 5th instar larval stage. However, the protein concentration decreased significantly in 3rd (*F* = 9.81, 13.38, df = 9, *P* < 0.05), 4th (*F* = 56.04, 361.52, df = 9, *P* < 0.05) and 5th instar (2.0 mg/L chlorpyrifos) (*F* = 16.48, df = 9, *P* < 0.05) larvae of pesticide exposed groups compared to the control. In contrast, there was no significant difference in protein concentration of the control and 1.5 mg/L chlorpyrifos affected group of 5th instar larvae (*F* = 6.76, df = 9, *P* > 0.05). Furthermore, in the both sub lethal concentrations the trehalose concentration decreased significantly in 3rd (*F* = 18.47, 60.95, df = 9, *P* < 0.05) and 5th instar (*F* = 116.53, 63.09, df = 9, *P* < 0.05), while there was no significant difference between control and treated groups of 4th instar stage (*F* = 0.27, 1.06, df = 9, *P* > 0.05). Similar result was obtained for lipid, where the concentration decreased significantly in 3rd (*F* = 44.18, 36.64, df = 9, *P* < 0.05) and 5th (*F* = 202.83,39.89, df = 9, *P* < 0.05) instar larvae but there was not any significant change in 4th instar control and chlorpyrifos affected groups (*F* = 4.49, 7.69, df = 9, *P* > 0.05).

**Table 2:** **Comparison of bio chemical parameters in control and chlorpyrifos exposed *P. ricini* in different larval stages**.

	**3rd Instar**	**4th Instar**	**5th Instar**
**TREHALOSE CONCENTRATION (MG/G OF BODY WEIGHT)**			
Control	1.77 ± 0.02_a_	1.99 ± 0.02_a_	3.21 ± 0.05_a_
Chlorpyrifos (1.5 mg/L)	1.55 ± 0.08_b_	1.91 ± 0.20_a_	2.26 ± 0.14_b_
Chlorpyrifos (2.0 mg/L)	1.42 ± 0.07_c_	1.82 ± 0.28_a_	1.85 ± 0.29_c_
**PROTEIN CONCENTRATION (MG/G OF BODY WEIGHT)**			
Control	21.23 ± 0.72_a_	55.51 ± 1.7_a_	64.32 ± 0.89_a_
Chlorpyrifos (1.5 mg/L)	19.98 ± 1.90_a_	42.75 ± 1.35_b_	61.83 ± 1.05_a_
Chlorpyrifos (2.0 mg/L)	18.11 ± 1.24_c_	33.75 ± 0.85_c_	59.32 ± 2.0_b_
**LIPID CONCENTRATION (MG/G OF BODY WEIGHT)**			
Control	41.98 ± 2.72_a_	80.45 ± 0.90_a_	84.32 ± 0.27_a_
Chlorpyrifos (1.5 mg/L)	31.47 ± 0.30_b_	75.72 ± 3.10_a_	79.06 ± 0.57_b_
Chlorpyrifos (2.0 mg/L)	29.75 ± 2.12_c_	72.03 ± 2.10_a_	76.91 ± 1.0_c_

Values not sharing a common subscript letter within each column differ significantly at P < 0.05 level.

The experiments were performed three times in triplicates (n = 10).

### Enzyme activity study

**Figure 2:**
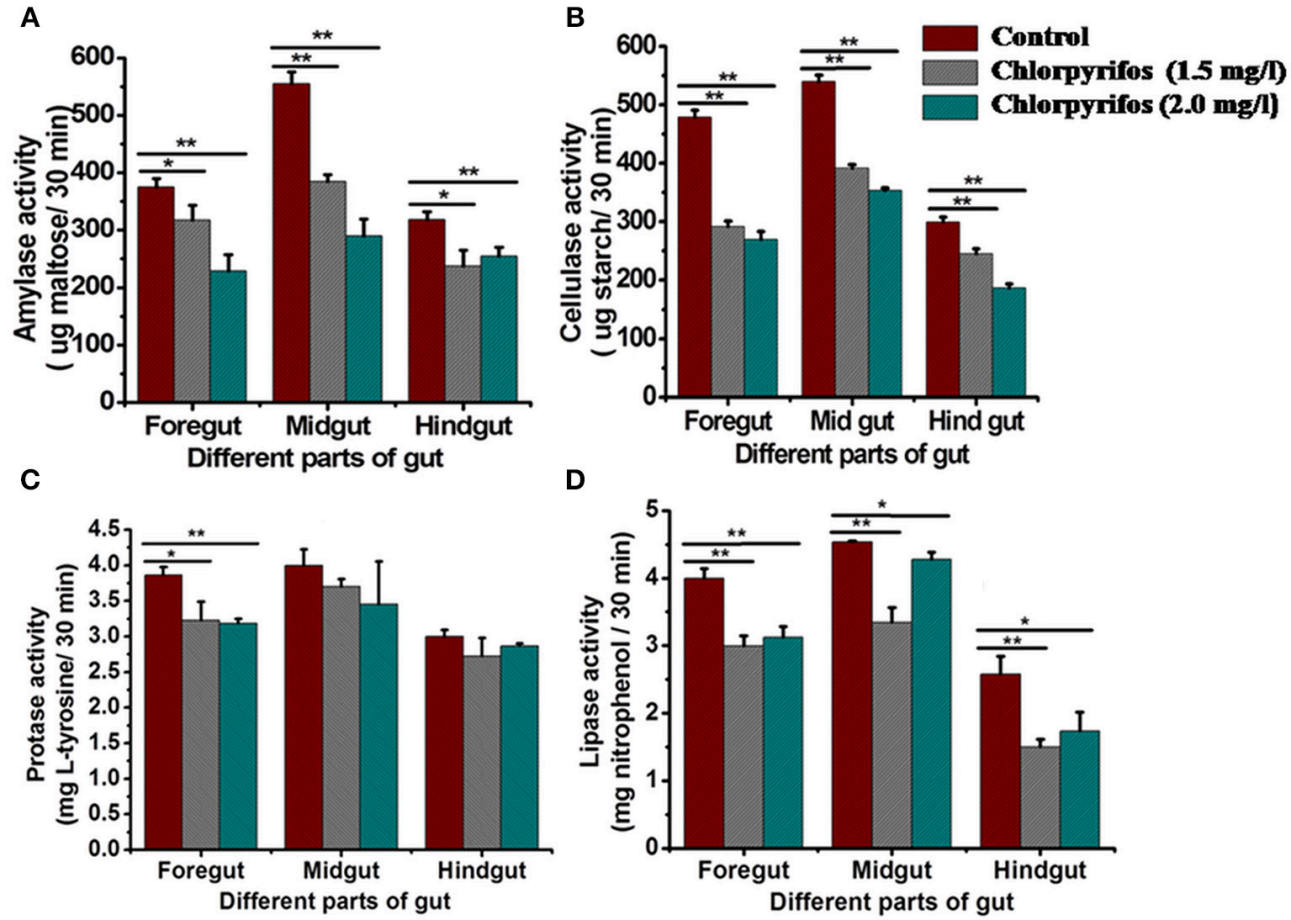
**Digestive enzyme activity indifferent parts of the gut of control and chlorpyrifostreated *P. ricini* larvae after 24 h of the exposure period. (A)** Amylase **(B)** Cellulase **(C)** Protease and **(D)** Lipase (^*^P < 0.05, ^**^P < 0.01 level of significance). All the experiments for enzyme assay were performed three times in triplicates (*n* = 10).

**Figure 3:**
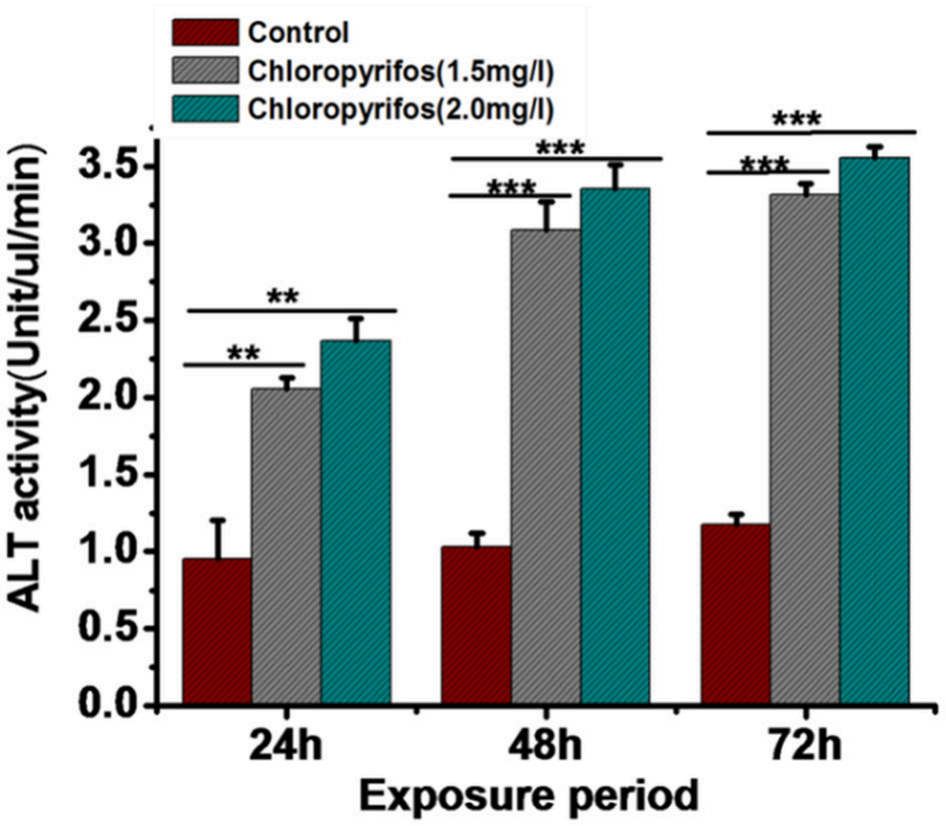
Comparison of alaninaminotransferase activity in control and chlorpyrifos treated groups of *P. ricini* in different time intervals (^**^P < 0.01, ^***^P < 0.001 level of significance). The experiment was performed three times in triplicates (*n* = 10).

Digestive enzyme activities were tested in chlorpyrifos exposed (1.5 and 2.0 mg/L) and control group to observe the effect of pesticide on digestive physiology of the eri silkworm. All the different parts of gut showed different digestive enzyme activity (amylase, cellulase, protease, and lipase). The enzyme activity was highest in midgut followed by foregut and hindgut for all the enzymes. The amylase, cellulose, and lipase activity was significantly lower in both of the treated groups than the control group in all parts of the gut (Figures 2A,B,D, Foregut: *F* = 11.26, 63.23, 434.68, 387.57, 70.75, 49.9, Midgut: *F* = 149.21, 165.94, 400.25, 713.02, 84.86, 17.79, Hind gut: *F* = 20.33, 28.3, 58.17, 307.28, 41.56, 14.45, *P* < 0.001). However, in both of chlorpyrifos exposed groups protease enzyme activity did not show any significant difference with the control batch except forgut (Figure 2C, Foregut: *F* = 15.41, 87.9, *P* < 0.05 Midgut: *F* = 4.6, 2.19 *P* > 0.05 Hind gut: *F* = 3.14, 6.34 *P* > 0.05). Further, the ALT enzyme activity also showed alteration and an increasing trend in ALT activity was observed in pesticide treated groups at different time intervals (24 h: *F* = 53.87, 90.59; 48 h: *F* = 295.55, 492.15; 72 h: *F* = 1281.16, 1572.95, df = 9, *P* < 0.001) (Figure 3).

### Histopathological study

**Figure 4:**
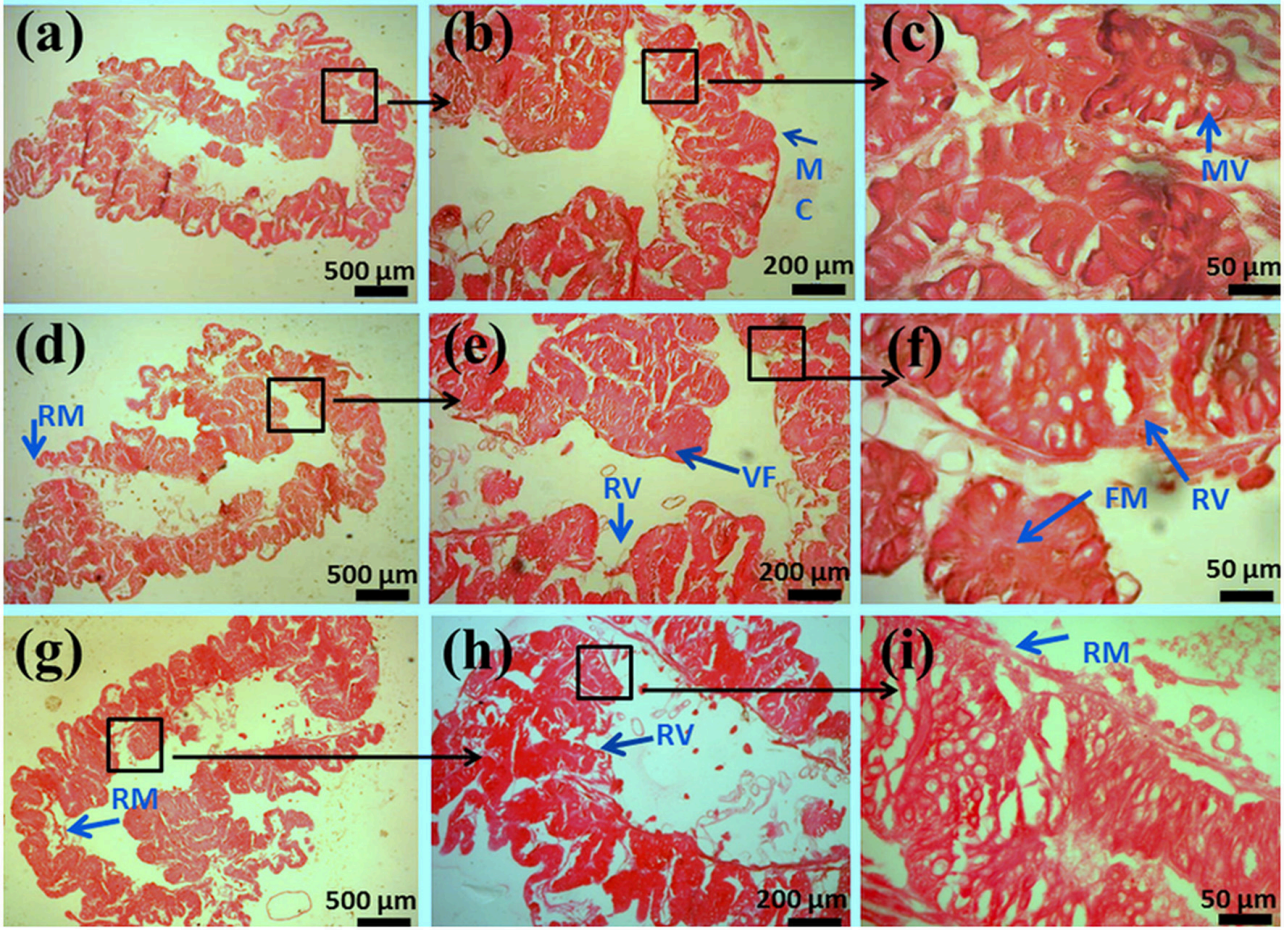
**Histopathology of the midgut in the fifth-instar larvae of *P. ricini* under chloropyrifos stress 24 h**. Control **(A)** 5x, **(B)** 10x, and **(C)** 40x resolutions showing normal structures of MC- mucosa, MV- microvilli. Treated gut tissues with chlorpyrifos (1.5 mg/L) **(D)** 5x, **(E)** 10x, and **(F)** 40x resolutions showing damaged structures RM- ruptured mucosa, RV- ruptured villi, VF- vacuole formation, FM- fused microvilli. Treated gut tissues with chlorpyrifos (2.0 mg/L) **(G)** 5x, **(H)** 10x, and **(I)** 40x resolutions showing damaged structures RM- ruptured mucosa, RV- ruptured villi, VF- vacuole formation, FM- fused microvilli. The experiment was performed three times in triplicates (*n* = 10).

In the control group, the mucosa and microvilli were intact and smooth in structure. Degenerative changes in the microvilli lining were observed in chlorpyrifos treated groups (1.5 and 2.0 mg/L). Additionally, thin mucosa, broken, and fused microvilli and vacuolation were observed along with mild hemorrhage in gut tissue treated with sublethal doses of chlorpyrifos (Figure 4).

### Genotoxicity assay

#### Alkaline single cell gel electrophoresis

**Figure 5:**
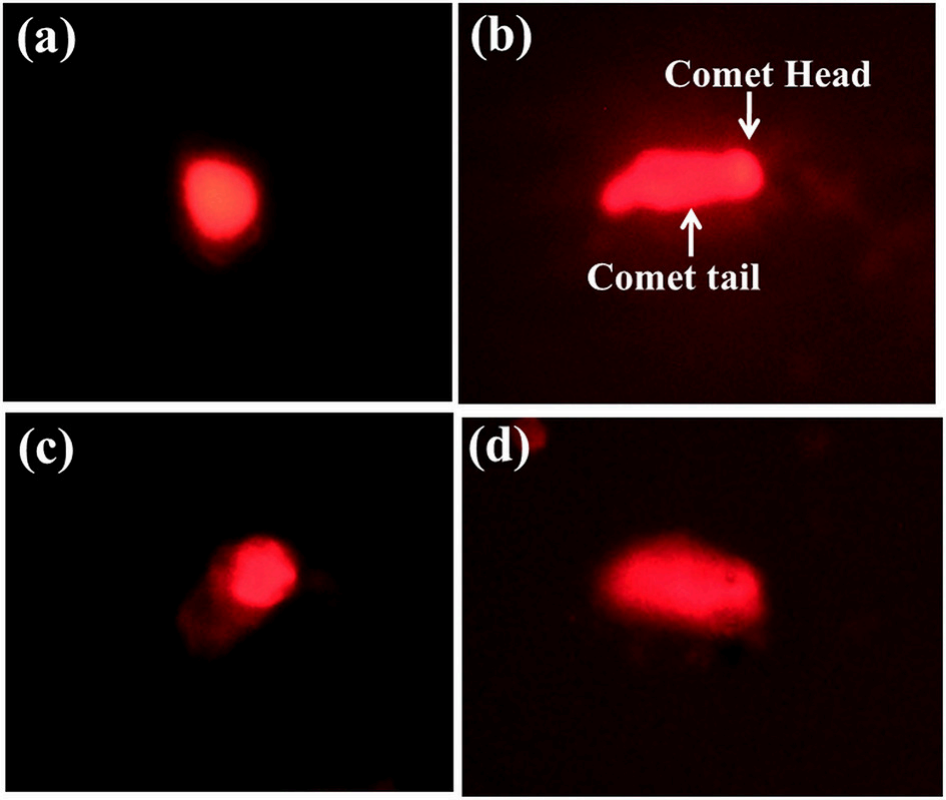
**Single cell gel electrophoresis images of *P. ricini* hemocytes (40x) (A)** control hemocyte with intact DNA, **(B)** DNA damage in positive control group treated with H_2_O_2_, **(C)**, and **(D)** DNA damage in hemocyte after treated with chlorpyrifos (1.5 and 2.0 mg/L respectively). The experiment was performed three times in triplicates (*n* = 10).

The effect of the sub lethal concentrations of chlorpyrifos on genotoxicity was observed within 24 h of exposure. The visual scoring analysis was performed to studythe DNA damage percentage. DNA damage was analyzed by comparing the tail length and tail DNA percentage of the control and positive control (treated with H_2_O_2_) batch. The control group showed round comet with intact nucleus (Figure 5A). In the positive control group treated with H_2_O_2_, the comets were elongated in structure with a long tail, which indicated DNA damage (Figure 5B). As shown in Table 3, significantly higher level of DNA damage was detected in silkworm with sub lethal concentrations of chlorpyrifos as compared with the corresponding controls. With increased sub lethal concentrations of chlorpyrifos, the tail DNA percentage (*F* = 2221.94, 7809.41, df = 9, *P* < 0.001) and tail length (*F* = 430.82, 1741.40, df = 9, *P* < 0.001) were increased significantly. At 2.0 mg/L concentration, almost 23 fold increases in tail length was observed and the hemocytes showed similar types of comets as H_2_O_2_ treated groups (Figures 5C,D).

**Table 3:** **DNA damage in the hemocytes of *P. ricini* with different sub lethal concentrations of chlorpyrifos after 24 h of exposure period**.

	**Head DNA (%)**	**TailDNA (%)**	**Taillength (μm)**	**Tailmoment (Arbitrary unit)**
**Control**	92.83 ± 4.20_a_	7.16 ± 4.11_a_	1.99 ± 0.65_a_	0.14 ± 0.01_a_
**Positive control**	23.59 ± 4.10_b_	76.40 ± 4.10_b_	20.08 ± 1.60_b_	15.36 ± 1.72_*b*_
**1.5 mg/L**	36.16 ± 6.49_c_	63.83 ± 6.49_c_	11.27 ± 2.48_c_	7.18 ± 1.63_c_
**2.0 mg/L**	25.17 ± 2.41_d_	74.66 ± 2.46_d_	19.97 ± 1.40_d_	14.90 ± 1.07_d_

The experiment was performed three times in triplicates (n = 10). Values not sharing a common subscript letter within each column differ significantly at P < 0.001 level.

#### Micronuclei assay

**Figure 6:**
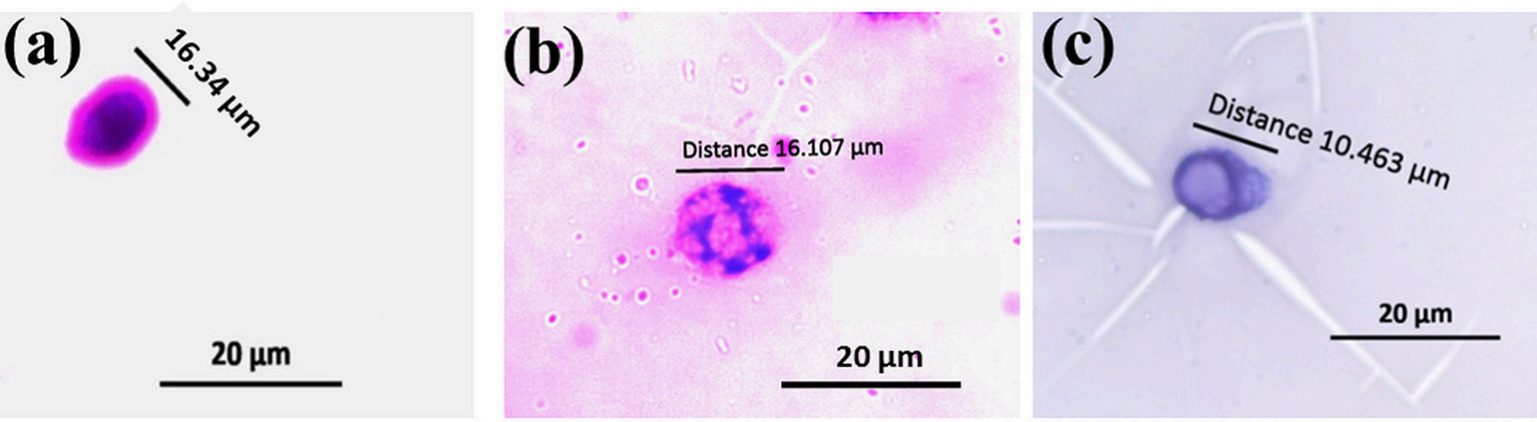
**Micronuclei formation in hemocytes of *P. ricini* after 24 h exposure to chlorpyrifos (20x) (A)** normal nuclear cell, **(B)**, and **(C)** cell with micronucleus in chlorpyrifos affected groups (1.5 and 2.0 mg/L respectively). The experiment was performed five times in triplicates (*n* = 5).

Visual scoring of micronuclei was performed by counting 1000 cells per slide, based on the criteria of clearly separable smaller nuclei from the main nucleus by phase contrast microscope. Hemocytes which showed intact nucleus were of control silkworms (Figure 6A), however the treated groups showed visible micronuclei (Figures 6B,C). Silkworms exposed to sub lethal concentration of chlorpyrifos showed a significant increase in micro nucleated hemocytes at 24 h exposure (*F* = 1012.19, 3685.29, df = 4, *P* < 0.001). The obtained data revealed a dose-dependent increase in micronuclei formation in chlorpyrifos affected silkworm. A fold increase of 6 and 9 in micronuclei percentage was observed at 1.5 and 2.0 mg/L concentration respectively (Table 4).

**Table 4:** **Percentage of micronuclei formation in hemocytes of *P. ricini* exposed to different sub lethal concentrations of chlorpyrifos after 24 h exposure period**.

**Exposure Dose (mg/L)**	**Percentage of micronuclei (%)**
**Control**	7.50 ± 0.50_a_
**1.5 mg/L**	42.40 ± 1.83_bbb_
**2.0 mg/L**	63.83 ± 1.52_ccc_

The experiment was performed five times in triplicates (n = 5). Values not sharing a common subscript letter within each column differ significantly at P < 0.001 level.

#### Caspase activation assay

**Figure 7:**
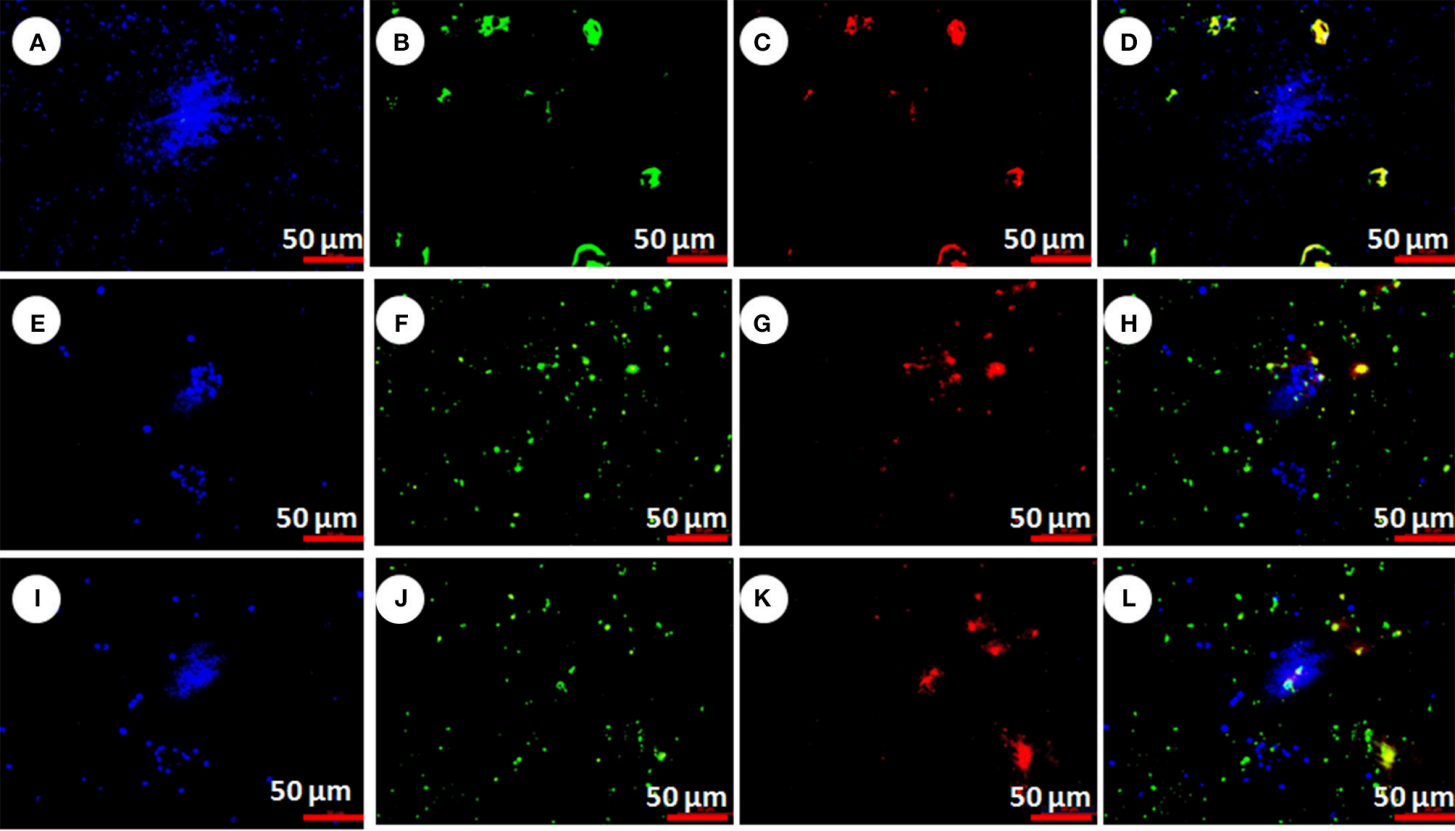
**Chlorpyrifos induced hemocyte death in *P. ricini* after 24 h exposure**. Control hemocytes with live cells, dead cells, apoptotic cells and merged pictures (**A–D** respectively); chlorpyrifos exposed group 1.5 mg/L (**E–H** respectively) and chlorpyrifos exposed group 2.0 mg/L (**I–L** respectively). The experiment was performed three times in triplicates (*n* = 10).

Caspase activation (procaspase to active caspase) elucidates the mechanism of cell death. In present study the active caspase was detected by FAM-DEVD-FMK caspase reagent (FLICA). After staining live cells showed blue color (stained with Hoechst 33,342), caspase activation showed green color (stained with FLICA reagents) and apoptotic cells showed red color (stained with PI). The live and dead cells were showed in Figure 7. The numbers of live cells were significantly higher in control group (Figure 7A) in comparison to pesticide treated groups (Figures 7B,C, *F* = 10629.4, *F* = 15199.2, df = 9, *P* < 0.001). However, in pesticide exposed groups significantly higher caspase activated and apoptotic cells (Figures 7F,G,J,K) were observed than live cells (Figures 7E,I) compared to control (*F* = 2987.5, *F* = 4931.2, *F* = 2352.4, *F* = 1958.1, df = 9, *P* < 0.001).

#### Annexin V affinity assay for detection of apoptotic hemocytes

**Figure 8:**
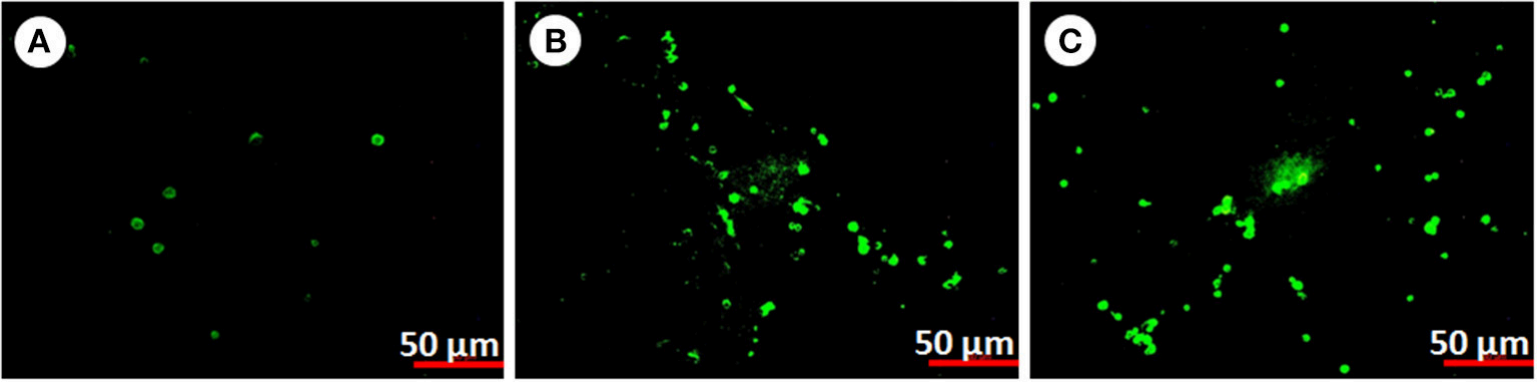
**Apoptosis in *P. ricini* hemocytes (A)** control, **(B)** chlorpyrifos exposed group (1.5 mg/L) and **(C)** chlorpyrifos exposed group (2.0 mg/L) after 24 h of exposure. The experiment was performed three times in triplicates (*n* = 10).

In order to confirm apoptosis in hemocytes exposed to pesticides we perform the annexin V affinity assay (Figure 8). Annexin V is widely used to detect apoptosis as it has a high affinity to phosphatidylserine, which is translocated from the inner to the outer plasma membrane of apoptotic cells. Both control and pesticide exposed hemocytes were stained with Alexa FluorR 488 annexin V and PI. In control group significantly less numbers of apoptotic cells were detected compared to both pesticide exposed groups (Figures 8B,C, *F* = 2659.6, *F* = 2759.5, df = 9, *P* < 0.001).

## Discussion

The average use of pesticide in Assam is comparatively lesser than national average (Krishijagaran, 2014^1^; Compedium of Environment Statistics 2008–2009^2^^,^^3^); however use of heavy doses of chlorpyrifos has been observed in the tea estate. In this study the acute toxicity of chlorpyrifos to eri silkworm was evaluated in terms of behavioral, biochemical and physiological alteration and also focused on the induced genotoxic effect of chlorpyrifos on silkworm. The present investigation showed a concentration-dependent increase in lethality which indicates that mortality was directly proportional to chlorpyrifos concentration. The time dependent decrease in LC_50_ values signifies that lethal concentration was inversely proportional to the exposure period. For a short exposure period (24 h), to obtain 50% mortality relatively higher concentration of chlorpyrifos is needed than a longer exposure period (96 h). Additionally, toxicity symptoms like body blackening, vomiting and body shaking occurred in chlorpyrifos affected larvae, which might be due to the neurotoxic effect of organophosphate pesticide leading to nervous breakdown of the organism (Zhang et al., 2008; Wang et al., 2015). The acute toxicity of different organophosphates was evaluated on *B. mori* and the intoxication symptoms caused by these pesticides are in agreement with the present result; however the LC_50_ values were not in the similar range (Zhang et al., 2008). The LC_50_ value of chlorpyriphos in *B. mori* recorded for 96 h as 3.45 mg/L which was less toxic compared to *P. ricini* (2.35 mg/L) of our study (Yu et al., 2016). Moreover, LC_50_ value for organophosphate phoxim in *B. mori* at 24 and 48 h were recorded as 1.05 and 0.45 mg/L respectively, whereas for dichlorvos the LC_50_ values were 6.63 and 4.11 mg/L. The variation in toxicity of organophosphates might be due to various factors such as different silkworm species as well as physiological and environmental factors (Zhang et al., 2008). Also recorded the LC_50_ value of phoxim on *B. mori* and wild *B. mandarina* silkworm as 2868.8 and 11543.9 ng/ml and interestingly it is reported that the wild silkworms were more resistant to pesticide. Hence, it might be assumed that the genetic factor play an important role in pesticide resistance mechanism in organisms.

Although the lethal concentration of pesticide induced toxicity in organisms, however the concentration below the LC_50_ value might also have some toxicological effect onorganisms. A significant decrease in biochemical constituents was observed in the larvae when exposed to sub lethal concentration of chlorpyrifos. The larvae which were fed on pesticide contaminated leaves showed less feeding behavior, thus their nutritional requirement was not fulfilled. Etebari et al. (2005) suggested that the starvation or less feeding could cause reduction of many biochemical compounds, therefore it might be considered as an important factor for decrease in all biochemical constituents. Moreover, Rharrabe et al. (2008) observed a significant decrease in protein, glycogen and lipid contents on exposure to azadirachtin in *P. interpunctella* Hübner. The reduction of such biochemical contents might be due to the absence of nutrients which is caused by the toxic effect of azadirachtin on the midgut. The present results are in agreement with the earlier studies, which reported a significant decrease in total protein, carbohydrate and cholesterol concentration in *B. mori* larvae, when exposed to sub lethal doses of various pesticides (Nath et al., 1997; Nath, 2000; Etebari et al., 2007). The depletion of protein in tissue might be due to the compensatory mechanism of insects during insecticidal stress condition. Under these circumstances, the protein molecules generally break into amino acids and enter into the trichloroacetic acid (TCA) cycle as keto acid and supply energy to the insect (Nath et al., 1997). Trehalose is considered as the blood glucose of insects and hence any alteration in trehalose concentration to meet the energy demand under stress condition can be expected (Thompson, 2003). Studies on *B. mori* exposed to pesticide also showed decrease in glucose concentration with increased exposure time. This hypoglycemia might be due to accelerated glycolysis or transportation of metabolite from the fat body to hemolymph in order to compensate energy crisis during stress condition (Nath, 2002). Lipids are the important source of energy in insects and lipid turn over in insects is regulated by neuroendocrine-controlled feedback loops (Downer, 1985). The pesticide exposure might impair the lipid synthesis mechanism of insects, leading to the depletion in lipid concentration.

Digestive enzymes also play an important role in growth and development of insects. The process of digestion and the activity of enzymes depend on different aspects such as the quantity and quality of ingested food, age and health of the larva and some physical factors (Vyjayanthi and Subramanyam, 2002). Any disruption in enzyme activity disables insects to obtain their nutrients for biological requirements. According to Vyjayanthi and Subramanyam (2002) the decrease in enzyme activity was due to unavailability of substrate resulting from insufficient food intake. Feeding is extremely important for the stimulation of digestive enzyme secretion (Shekari et al., 2008). In this study, the chlorpyrifos treated leaves fed larvae exhibited less feeding behavior, therefore the starvation or unavailability of substrate to form enzyme-substrate complex might be the reason for alteration in digestive physiology of the larvae. Similarly, Less feeding behavior after *Melia azedarach* a botanical insecticide treatment might have interfered with the enzyme—substrate complex thus affecting the peristaltic movement of the insect gut (Broadway and Duffey, 1988; Duffey and Stout, 1996) which was clearly observed by the decrease of fecal pellet production (Senthil-Nathan, 2006, 2013). Further, plant compounds used as insecticide might also affect enzyme titers and activities as demonstrated by Klocke (1989) and Feng et al. (1995). Additionally, mid gut of Lepidopteran insect possess endocrine cells which (Endo and Nishiitsutsuji-Uwo, 1980) might responsible for local control of enzyme secretion into the gut lumen. Azadirachtin may affect the secretory function of neuroendocrine cells in insects which is followed by inhibition of enzyme activity (Barnby and Klocke, 1990; Garcia et al., 1990). The inhibition of secretory function of neuroendocrine cells of *P. ricini* due to the effect of chlorpyrifos might be another reason for lower activity of enzymes. However, protease activity of chlorpyrifos affected larvae did not show any significant changes. Nath et al. (1997) studied the protein metabolism system in *B. mori* and clearly indicated that the decrease in protein concentration was associated with elevated protease activity. We have observed that in the chlorpyrifos exposed groups despite of significant decrease in protein concentration, the alteration in protease activity was not significant.

The histology study of the gut of pesticide exposed silkworm clearly indicated the degenerative changes in gut lining. The damage in gut structure might be considered as one of the reason for the impairment of the normal functioning of the digestive enzymes. Phoxim exposed group of *B. mori* silkworms also showed similar kind of results where loss of basal lamina and karyopyknosis in midgut tissue was reported as a result of pesticide poisoning (Su et al., 2014).

Alanineaminotransferase (ALT) enzyme activity is considered as an indicator for organophosphate poisoning in insects (Ender et al., 2005). The ALT serves as a strategic link between the carbohydrates and protein metabolism and is proved to be altered during various physiological and pathological conditions (Etebari et al., 2005). The activity level of ALT increased significantly in pesticide affected 5th instar larvae starting from 24 to 72 h. This result was in support of the hypothesis that an increase in lipid peroxidation leads to an increase in ALT activity. Elevation of ALT activity in pesticide exposed *B. mori* indicated an active transportation of amino acids which provide keto acid to serve as a precursor in the synthesis of essential constituents under stress condition (Nath et al., 1997).

Environmental pollutants, especially pesticides also have induced genotoxicity in organisms. Micronucleus assay has been used as a measure to assess genotoxicity in organisms caused by different pesticides under laboratory as well as field condition (Lajmanovich et al., 2005; Yin et al., 2009; Botelho et al., 2015). The formation of micronuclei was observed in pesticide affected groups of the silkworm and the rate of micro nuclei formation was also increased with the increased sub lethal doses of chlorpyrifos. The potential molecular mechanism of organophosphates primarily includes induction of alkylation on DNA level. Being an alkylating agent, this might cause disturbance in DNA replication procedure leading to abnormal nuclear division and micronuclei formation. Moreover, micronuclei assay is an efficient tool to detect the clastogenic and anegenic compounds in environment. The substances, which form large micronuclei, are called aneugens and the small micronuclei are formed by clastogens (Vanderkerken et al., 1989). During the study smaller micronuclei formation was observed, therefore clastogenic effect of chlorpyrifos might be expected in the exposed groups.

Single cell gel electrophoresis is another highly sensitive technique and used as a marker to evaluate DNA damage in organisms (Fairbairn et al., 1995). The technique is based on the fact that the damaged DNA can migrate more easily in an electric field than the intact one. The cells appear as a comet with a head region containing undamaged DNA and a tail of damaged DNA. The severity of DNA damage is directly correlated with the migration distance (Singh et al., 1988). In the present study, chlorpyrifos act as a genotoxicity inducing agent by exhibiting relatively longer tail region at 24 h of treatment and the severity of DNA damage was dose dependent. Since organophosphates are the potential alkylating agents, the protein alkylation may be directly or indirectly involved in DNA disintegration. Moreover, the phosphorus moiety of organophosphate is a favorable substrate for nucleophilic attack, which causes phosphorylation of the DNA leading to DNA damage (Wild, 1975). As reported by Porichha et al. (1998) tail formation is also an indicative of apoptosis, where apoptotic cells show nuclear fragmentation and occurs as a tail. These findings are in agreement that organophosphates cause genotoxicity in a wider range of non-targets organisms (Porichha et al., 1998; Rahman et al., 2002; Altinok et al., 2012). Moreover, pesticide inducing DNA damage on *B. mori* larvae were also evaluated and a positive correlation between pesticide concentration and DNA damage was reported (Yin et al., 2008; Shen et al., 2011). Since, the hemocytes are considered as the key energy source of insects and play a role in the compensatory mechanism during growth period; any impairment of hemocytes in terms of genotoxicity might induce negative impact on the growth and development of the silkworm.

The DNA damage might be considered as a trigger for apoptosis, however for validation of whether the damage caused by chlorpyrifos is due to apoptosis we extended our study. Herein, we investigated the molecular mechanisms by which exposure to chlorpyrifos induces DNA damage in silkworm. Results showed that chlorpyrifos induced apoptosis in silkworm hemocytes and initiated a series of cell death signaling event including activation of caspase leading to DNA fragmentation. To our knowledge this is the first report of chlorpyrifos induced apoptosis in silkworm *in vivo*. However, chlorpyrifos induced apoptosis was also observed in *Drosophila* larvae, human monocyte cell line U937 and human placental carcinoma cells (Nakadai et al., 2006; Saulsbury et al., 2008; Gupta et al., 2010). To elucidate the mechanism of chlorpyrifos induced apoptosis we performed caspase assay and annexin V affinity assay. Caspases are initially expressed as inactive procaspase but during cell death process they get activated leading to initiation of a protease cascade. As a result of this cascade one caspase activates other caspases and once caspase gets activated it seems to be an irreversible commitment to cell death process. Likewise, annexin V also serves as an indicator of apoptosis as it is a recombinant phosphatidylserine-binding protein that interact strongly and specifically with phosphatidyl serine residues of dead cells (Elmore, 2007). It is used in conjunction with vital dyes which bind to nucleic acids, but can only penetrate the plasma membrane when membrane integrity is breached which occurs in later stages of apoptosis or in necrosis (Heerde et al., 2000). According to Gupta et al. (2010) caspases might be the target of chlorpyrifos along with other factors leading to cell death of silkworm. Similarly chlorpyrifos might disturb the plasma membrane integrity of silkworm hemocytes followed by DNA fragmentation and as a result apoptosis signals were generated.

The organophosphate insecticide chlorpyrifos has a wide range of effects. The present study showed two aspects firstly the effect of chlorpyrifos on nutritional physiology and secondly induced genotoxicity on *P. ricini*. The exposure of sub-lethal concentration of chlorpyriphos showed behavioral changes in terms of less feeding which lead to the reduction of metabolite content along with inhibition of digestive enzymes activity. The alteration of this nutritional physiology might be regarded as a stress response due to unavailability of required nutrition leading to alteration in enzyme-substrate complex and followed by inhibition of digestive enzyme activity. Histology of silkworm gut supports the result of less nutritional supply as the gut villi cells are deformed and necrosis occurs they might not able to absorbed required nutritional molecules or due to destroyed gut lining not able to secrete the digestive enzymes properly. Additionally, the inhibition of secretory function of neuroendocrine cells of *P. ricini* due to the effect of chlorpyrifos might be another reason for lower activity of enzymes. Another study showed that the organophosphate insecticide chlorpyrifos induce micronucleus formation, DNA damage followed by apoptosis in *P. ricini*. All these aspects are interrelated, chlorpyrifos is a strong alkylating agent bound to the DNA of sister chromatids and due to non disjunction of chromosomes a small fragment of DNA appeared as micronuclei. This is followed by single or double DNA breaks by the action of chlorpyrifos and appeared as a comet in the comet assay. Due to DNA fragmentation the signals of apoptosis are generated, caspase get activated and the cell undergoes apoptosis. Further extension of the work is needed for the molecular mechanism of stress response pathway induced by chlorpyrifos.

Considering the negative effect of the sub lethal concentration of chlorpyrifos on eri silkworm, there should be a serious apprehension about the potential danger of this pesticide on silkworm. This study might be helpful for the safe and judicial use of pesticide in agricultural practices, so that it may not cause serious loss to non-targeted species like the silkworm and sericulture industry.

## Author contributions

MK design the work, carry out the experiments and draft the manuscript. KH helps in the experiments, analysis and manuscript preparation. DD supervise the work and helps in manuscript preparation.

### Conflict of interest statement

The authors declare that the research was conducted in the absence of any commercial or financial relationships that could be construed as a potential conflict of interest.

## Footnotes

^1^“http://krishijagran.com/corporate-watch/Industry-Profile/2014/11/Outlook-of-Pesticide%20Consumption-in-India.”

^2^“http://mospi.nic.in/Mospi_New/upload/Compendium_of_Environment_Statistics%20_2008_09/comp_env_2008-09_chp5.pdf.”

^3^“http://www.planassam.info/economic_survey_assam_13-14/Economic_Survey_%202013-14.pdf.”
